# Evaluation of Cardiovascular Disease Risk According to the Morningness-Eveningness Status in Adult Individuals

**DOI:** 10.7759/cureus.86751

**Published:** 2025-06-25

**Authors:** Ayfer Beyaz Coskun, Emine Elibol, Sehriban Duyar Ozer

**Affiliations:** 1 Nutrition and Dietetics, Firat University, Elazig, TUR; 2 Nutrition and Dietetics, Ankara Yıldırım Beyazıt Üniversitesi, Ankara, TUR; 3 Nutrition and Dietetics, Hitit University, Corum, TUR

**Keywords:** anthropometry, biochemistry, cardiovascular diseases, chronotype, exercise, morningness-eveningness status, sleep

## Abstract

Background

Chronotype is the individual's tendency to exhibit morning or evening behaviors. Having an evening chronotype creates an environment for different chronic diseases. An individual's lifestyle behaviors that are contrary to the endogenous circadian rhythm pave the way for hypertension and other cardiovascular diseases (CVDs).

Methods

This study, which was conducted to evaluate the relationship between chronotype and CVD risk in adult individuals, involved a total of 1,100 individuals aged between 19 and 64 years who applied to a family health center in Elazig, Turkey. The demographic questionnaire, the self-assessment Morningness-Eveningness Questionnaire (MEQ), and the Framingham Risk Score were used to collect data. Biochemical parameters were queried from patient records with measurements taken in the last week, and anthropometric measurements were recorded by researchers.

Results

At the end of the study, it was determined that individuals in the eveningness group had a higher risk (N=31, 29.8%) of CVDs. In addition, the percentage of individuals with body mass index (BMI) (N=67, 64.4%), fasting blood glucose (FBG) (N=19, 18.3%), total cholesterol (N=44, 42.3%), and low-density lipoprotein cholesterol (LDL-c) (N=38, 36.5%) values ​​above the reference ranges was found to be higher in the evening chronotype group.

Conclusion

As a result, individuals with an evening chronotype are at higher risk for CVDs. In addition, obesity and some biochemical parameters are more common in individuals with an evening chronotype. Supporting healthy lifestyle activities by taking into account the chronotype habits of individuals may contribute to the prevention of chronic diseases.

## Introduction

Morningness or eveningness reflects the time periods that an individual prefers to perform various activities such as eating, working, sleeping, and exercising [1]. This chronotype provides insight into lifestyle patterns, influences the circadian rhythm, and is indirectly linked to various health conditions [2-4]. Among these are chronic diseases strongly affected by lifestyle factors, including obesity, diabetes, cardiovascular diseases (CVDs), and sleep disorders [5].

CVDs, which are important determinants of morbidity and mortality, are a group of diseases that require medical treatment worldwide and have a high healthcare burden [6]. Many metabolic disorders are seen together in the etiology of CVD, and the risk of stroke increases with age [7]. Various factors such as genetics, age, gender, smoking status, and the presence of chronic diseases pose a risk for CVD [8]. Calculating the risk before the disease occurs and preventing or delaying it with primary healthcare services is of great importance for the protection of society and the reduction of CVD [9].

Given the high prevalence of CVD [10] and its association with lifestyle, this study aims to evaluate CVD risk in adults based on their morningness-eveningness status. It is hypothesized that predisposition to CVD may vary according to chronotype. Additionally, this study's originality lies in its comprehensive approach by integrating anthropometric measurements with chronotype analysis to better understand cardiovascular risk.

## Materials and methods

This study was conducted on a voluntary basis with adult individuals. It was approved by the Non-interventional Research Ethics Committee of Firat University, Elazig, Turkey, with the decision numbered 2024/08-14 to comply with ethical rules. All procedures were performed in accordance with the Declaration of Helsinki. Before the study, participants were informed about the purpose and method, and then a consent form was signed by the individuals.

Type of research

This study was planned as a descriptive and cross-sectional study to evaluate the relationship between the morningness-eveningness status of individuals and heart disease.

Location and sample of the research

The study was conducted between August 2024 and February 2025 with a total of 1,100 individuals aged between 19 and 64 years who applied to five different family health centers in Elazig, Turkey. A sample size calculation was performed for our study, drawing on data from a similar reference study by Makarem et al. [11]. Using these data, a power analysis was conducted to determine the required sample size for our study, targeting a similar effect size (Cohen’s f ≈ 0.25) at an alpha level of 0.05 and a power of 0.95. The analysis indicated that a total sample size of approximately 960 participants would be sufficient to achieve robust statistical power for detecting significant differences across groups [11]. Therefore, with a planned sample size of approximately 1,050 participants, our study is designed to ensure reliable and comprehensive findings across the chronotype and dietary quality quartiles. Randomly selected participants were included in the study on a voluntary basis. Individuals who were previously diagnosed with any sleep disorder by a doctor and for whom patient records could not be obtained within the recommended time period were not included in the study.

Data collection tools

Demographic questionnaire, self-assessment Morningness-Eveningness Questionnaire (MEQ), and Framingham Risk Score were used to collect data. Biochemical parameters were queried from patient records with measurements taken in the last week (since they were up-to-date), and anthropometric measurements were recorded by researchers. Permissions for the use of all scales mentioned in the article have been obtained.

Demographic information

A total of 13 questions were asked through the demographic information questionnaire, such as participants' gender, age, and smoking (Appendix A).

Anthropometric measurements and biochemical parameters

The individuals' height was measured, and their body weights were measured using the Tanita BC 601 device (Tanita Health Equipment H.K. Limited, Tokyo, Japan). Body weight measurements were made at any time of day following at least four hours of fasting. Body mass index (BMI) was calculated using body weight and height. In addition, the participants' waist and hip circumferences were measured with a non-stretchable tape measure, and then the waist/hip ratio was calculated. The participants' high-density lipoprotein cholesterol (HDL-c), low-density lipoprotein cholesterol (LDL-c), total cholesterol, and fasting blood glucose (FBG) values ​​were taken from the biochemical parameters results of the patients in the last week, and no extra blood was taken from the patients. Blood samples collected from family health centers were examined in a single central hospital.

Self-assessment MEQ

The scale used to determine morningness and eveningness, consisting of a total of 19 questions, consists of four options (Appendix B) [12]. The answers to questions one, two, and 10 are calculated using a ruler set according to a seven-hour time period. This ruler represents 15-minute time intervals. Scoring ranges from 16 to 86, with low scores indicating evening and high scores indicating morning. According to the total score obtained, individuals were classified as eveningness (score: 16-41), intermediate (score: 42-58), and morningness (score: 59-86) types. The validity and reliability of the scale were examined by Pündük et al. (2005) [13].

Framingham risk score

The Framingham Risk Score, which was developed in North America and updated in Canada to calculate CVD risk [14, 15], has been proven to be valid for use in different nations and is also recommended by the Turkish Cardiology Association [16, 17]. The Framingham Risk Score evaluates the proximity of individuals to CVD according to their age, gender, smoking status, lipid profile, blood pressure, and diabetes (Appendix C)[18].

Framingham Risk Scores of individuals were calculated using the table published on the official website of the Turkish Cardiology Association. Individuals' age, smoking, gender, diabetes diagnosis, LDL-c and HDL-c values, and systolic and diastolic blood pressures were recorded in the system. Those with a 10-year CVD risk of 20% and above were grouped as high risk, those between 10% and 19% as medium risk, and those below 10% as low risk [16].

The three-question short form used by Marshall and his colleagues was applied to evaluate the physical activity status of the students. The first question asks about regular physical activity status. In the second question, "three or more activities per week" is evaluated as four points, "one to two times per week" as two points, and "never" as 0 points; in the third question, "five or more activities per week" is evaluated as four points, "three to four times per week" as two points, "one to two times per week" as one point, and "never" as 0 points. If the score obtained by adding the second and third questions is ≥4, it indicates that individuals are doing sufficient physical activity (Appendix D) [19].

Statistical analyses

Statistical analyses were performed using the IBM SPSS Statistics software, version 26 (IBM Corp., Armonk, NY). For comparing the values of two independent groups without a normal distribution, the Mann-Whitney U test (Z-table value) was used. For comparing the values of three or more independent groups without a normal distribution, the Kruskal-Wallis H test (χ²-table value) was applied. To examine the relationship between two quantitative variables that did not follow a normal distribution, Spearman's correlation coefficient was used.

## Results

A total of 1,100 individuals, 715 female and 385 male, were included in the study. The mean age of females was 39.7±13.35 years, the mean age of males was 42.5±13.03 years, and the total mean age was 40.69±13.3 years. Age, body weight, height, BMI value, waist-hip ratio, and waist and hip circumference of males were found to be statistically significantly higher than females (p<0.05) (Table 1).

**Table 1:** Mean value of anthropometric measurements of individuals according to gender BMI: body mass index; *values in bold denote statistical significance at the p < 0.05 level; γ: Mann-Whitney U test (Z value)

Variable	Female (n:715)	Male (n:385)	Total (n:1100)	p- value^γ^	Z value
	X±SD	X±SD	X±SS		
Age (years)	39.7±13.35	42.5±13.03	40.69 ± 13.3	0.001	-3.31
Body weight (kg)	83.3±16.29	92,2±14,51	86.4 ± 16.25	0.001	-8.42
Height (cm)	161.7±6.44	174.4±7.40	166.11 ± 9.09	0.001	-22.04
BMI (kg/m^2^)	32.0±6.42	30.4±4.68	31.42 ± 5.92	0.001	-3.89
Waist circumference (cm)	96.7±33.92	102.2±13.81	98.7 ± 28.52	0.001	-8.10
Hip circumference (cm)	111.7±13.83	112.4±42.86	111.91 ± 27.94	0.038	-2.13
Waist-hip ratio	0.86±0.10	0.94±0.11	0.88 ± 0.11	0.001	-11.96

Table 2 shows the number and percentage values ​​of individuals in the Framingham Risk Group according to the MEQ scale. Of the individuals in the morningness group, 31 (59.6%) were in the low-risk group, 12 (23.1%) were in the medium-risk group, and nine (17.4%) were in the high-risk group. Of the individuals in the intermediate type, 665 (70.4%) were in the low-risk group, 126 (13.4%) were in the medium-risk group, and 153 (16.2%) were in the high-risk group. Of the individuals in the eveningness group, 63 (60.6%) were in the low-risk group, 10 (9.6%) were in the medium-risk group, and 31 (29.8%) were in the high-risk group (chi-square, 0.002).

**Table 2:** Number and percentage values of individuals in the Framingham Risk group according to the self-assessment Morningness-Eveningness Questionnaire (MEQ) *Chi-square test

Variable		MEQ								
		Morningness		Intermediate		Eveningness		Total		χ2
		n	%	n	%	n	%	n	%	
Framingham Risk Group	Low	31	59.6	665	70.4	63	60.6	739	68.3	0.002
	Medium	12	23.1	126	13.4	10	9.6	150	13.9	
	High	9	17.4	153	16.2	31	29.8	193	17.8	

Table 3 shows the distribution of demographic, sleep duration, anthropometric, and biochemical parameters according to MEQ and Framingham Risk Group. Thirty-one (59.6%) of individuals in the morningness group were female, and 21 (40.4%) were male; 69 (66.3%) of individuals in the eveningness group were female, and 35 (33.7%) were male.

**Table 3:** Distribution of demographic, anthropometric, and biochemical parameters according to the self-assessment Morningness-Eveningness Questionnaire (MEQ) and Framingham Risk Group BMI: body mass index; HDL-c: high-density lipoprotein cholesterol; LDL-c: low-density lipoprotein cholesterol; F: female; M: male

Variable		MEQ						Framingham Risk Group					
		Morningness		Intermediate		Eveningness		Low		Medium		High	
		n	%	n	%	n	%	n	%	n	%	n	%
Gender	Female	31	59.6	615	65.1	69	66.3	489	64.4	75	50.7	151	78.2
	Male	21	40.4	329	34.9	35	33.7	270	35.6	73	49.3	42	21.8
Smoking	Yes	25	48.1	233	24.6	20	19.2	176	73.2	51	34.4	51	26.4
	No	27	51.9	711	75.4	84	80.8	583	26.8	97	65.6	142	73.6
Regular exercise	Yes	2	5.1	72	10.3	12	17.1	64	11.2	10	9.5	12	9.1
	No	37	94.9	625	89.7	58	82.9	505	88.8	95	90.5	120	90.9
Waist circumference (cm)	F≥ 80; M ≥94	42	84	754	84.1	77	76.2	591	82.5	118	83.7	164	85.9
	F< 80; M< 94	8	16	143	15.9	24	23.8	125	17.5	23	16.3	27	14.1
Waist-hip ratio	F ≥0.85; M ≥1	22	44.9	390	43.6	36	36.4	291	40.8	58	42	99	52.1
	F <0.85; M<1	27	55.1	504	56.4	63	63.6	423	59.2	80	58	91	47.9
BMI (kg/m^2^)	≥30	27	51.9	526	55.7	67	64.4	378	49.8	103	69.6	139	72
	<30	25	48.1	418	44.3	37	35.6	381	50.2	45	30.4	54	28
Hypertension	Yes	5	9.6	143	15.1	13	12.5	72	9.5	33	22.3	56	29
	No	47	90.4	801	84.9	91	87.5	687	90.5	115	77.7	137	71
Fasting blood glucose (mg/dL)	≥126	4	7.7	203	21.5	19	18.3	92	12.1	53	35.8	81	42
	< 126	48	92.3	741	78.5	85	81.7	667	87.9	95	64.2	112	58
Total cholesterol (mg/dL)	≥ 200	19	36.5	346	36.7	44	42.3	209	27.5	74	50	126	65.3
	<200	33	63.5	598	63.3	60	57.7	550	72.5	74	50	67	34.7
LDL-c (mg/dL)	≥ 130	13	25	366	38.8	38	36.5	242	31.9	59	39.9	116	60.1
	< 130	39	75	578	61.2	66	63.5	517	68.1	89	60.1	77	39.9
HDL-c (mg/dL)	F ≥50; M ≥40	28	53.8	504	53.4	52	50	439	57.8	84	56.8	61	31.6
	F <50; M <40	24	46.2	440	46.6	52	50	320	42.2	64	43.2	132	68.4
Sleep duration (hr)	≥8	30	57.7	446	47.2	55	52.9	345	45.5	77	52	109	56.5
	<8	22	42.3	498	52.8	49	47.1	414	54.5	71	48	84	43.5

The rate of individuals with hypertension was lower in the morningness group (N=5, 9.6%) than in the eveningness group (N=13, 12.5%). The rate of individuals with a BMI value of 30 kg/m² and above was 27 (51.9%) in the morningness group and 67 (64.4%) in the eveningness group. The rate of individuals with an FBG level of 126 mg/dL and above was four (7.7%) in the morningness group and 19 (18.3%) in the eveningness group. The rate of individuals with a total cholesterol level of 200 mg/dL and above was 19 (36.5%) in the morningness group and 44 (42.3%) in the eveningness group. The rate of individuals with an LDL-c value of 130 mg/dL and above was 13 (25.0%) in the morning group and 38 (36.5%) in the eveningness group. The majority of participants in both the morningness (N=30, 57.7%) and eveningness (N=55, 52.9%) groups slept eight hours or more per day.

According to the Framingham Risk Score, 489 (64.4%) of those in the low-risk group were female and 270 (35.6%) were male; 75 (50.7%) of those in the medium-risk group were female and 73 (49.3%) were male; and 151 (78.2%) of those in the high-risk group were female and 42 (21.8%) were male.

According to the Framingham Risk Score, the hypertension rate in individuals in the low-risk group was 72 (9.5%), while the hypertension rate in individuals in the high-risk group was 56 (29.0%). The rate of individuals with FBG levels of 126 mg/dL and above was 92 (12.1%) in the low-risk group and 81 (42.0%) in the high-risk group. The rate of individuals with total cholesterol levels of 200 mg/dL and above was 209 (27.5%) in the low-risk group and 126 (65.3%) in the high-risk group. The rate of individuals with LDL-c levels of 130 mg/dL and above was 242 (31.9%) in the low-risk group and 116 (60.1%) in the high-risk group. According to the Framingham Risk Score, the percentage of participants in the low-risk group who sleep eight hours or more per day was 345 (45.5%), while the percentage of those who sleep less than eight hours was 414 (54.5%).

Table 4 shows age, sleep duration, anthropometric and biochemical parameters, and MEQ and Framingham Risk Score groups. According to the morningness-eveningness scale, the average age of evening individuals was found to be statistically higher than morningness and intermediate types (p < 0.05). The lowest height and waist circumference were measured in the evening group in the study (p<0.05). The systolic blood pressure (124.26±34.96 mm/Hg) of individuals in the intermediate type was found to be higher than morningness and eveningness types (p<0.05). The morningness group had higher sleep hours on the weekend than the other groups (p<0.05). The Framingham Risk Score of individuals in the intermediate type was found to be 0.92±7.6, 2.06±7.31 for morningness, and 2.93±8.14 for eveningness (p<0.05).

**Table 4:** Anthropometric and biochemical parameter values according to the self-assessment Morningness-Eveningness Questionnaire (MEQ) and Framingham Risk Group *MEQ calculation was made for Framingham risk classification; BMI: body mass index; HDL-c: high-density lipoprotein cholesterol; LDL-c: low-density lipoprotein cholesterol; MEQ: self-assessment Morningness-Eveningness Questionnaire; SBP: systolic blood pressure; DBP: diastolic blood pressure; WC: waist circumference; HC: hip circumference; T-Chol: total cholesterol; The values in bold denote statistical significance at the p < 0.05 level; β: Kruskal-Wallis Test (x2 value)

	MEQ					Framingham Risk Group				
Variable	Morning^ 1^	Intermediate ^2^	Evening ^3^	p-value^β ^	X^2 ^ value	Low^1^	Medium^2^	High^3^	p-value^β^	X^2 ^ value
	𝐗±SD	𝐗±SD	𝐗±SD			𝐗±SD	𝐗±SD	𝐗±SD		
Age (years)	41.13 ± 13.73	40.07 ± 13.11	46.04 ± 13.68	0.001 (3-1,2)	17,71	34.67 ± 10.42	50.38 ± 7.47	56.92 ± 7.79	0.001 (1-2,3), (2-1,3), (3-1,2)	8.20
Body weight (kg)	84.92±14.16	86.66±16.46	85.23 ± 15.33	0.402	1,82	85.22 ± 157.73	111.57 ± 122.17	132.78 ± 188.17	0.001 (3-1,2)	9.39
Height (cm)	166.94 ± 9.49	166.34 ± 9.04	163.54 ± 9.07	0.012 (3-1,2)	8,84	166.99 ± 9.00	166.55 ± 8.92	162.28 ± 8.64	0.001 (3-1,2)	4.06
BMI (kg/m^2^)	30.62 ± 5.32	31.4 ± 5.94	32.01 ± 6.03	0.405	1,81	30.61 ± 5.71	33.08 ± 5.72	33.34 ± 6.18	0.001 (1-2,3)	11.90
WC (cm)	99.98 ± 14.27	98.08 ± 14.98	95.60 ± 13.46	0.042 (1-3)	2,59	98.55 ± 32.94	101.4 ± 15.84	97.09 ± 12.88	0.012 (2-1,3)	23.11
HC (cm)	111.06 ± 9.45	111.11±13.5	111.31 ± 13.65	0.973	0,06	111.84 ± 32.48	112.85 ± 13.62	111.42 ± 12.23	0.225	16.68
Waist-hip ratio	0.9 ± 0.09	0.89 ± 0.11	0.86 ± 0.12	0.153	3,76	0.88 ± 0.11	0.91 ± 0.1	0.88 ± 0.11	0.015 (2-1,3)	8.34
Fasting blood sugar (mg/dL)	165.62 ± 309.88	157.9±219.12	174.62±243.01	0.250	3,49	155.96 ± 231.38	160.81 ± 194.47	174.2 ± 229.59	0.001 (1-2,3)	12.6
T-Chol (mg/dL)	191.53 ± 39.00	192.36 ± 44.47	200.98 ± 52.39	0.302	3,61	183.96 ± 38.72	203.75 ± 47.29	221.06 ± 52.83	0.001 (1-2,3), (2-1,3), (3-1,2)	9.67
LDL-c (mg/dL)	116.92 ± 32.93	124.54 ± 43.1	125.00 ± 41.13	0.363	2,03	118.65 ± 40.90	128.89 ± 42.03	141.58 ± 44.38	0.049 (1-3)	6.62
HDL-c (mg/dL)	45.68 ± 9.11	48.08 ± 9.56	46.90± 9.60	0.418	1,75	48.95 ± 9.54	47.22 ± 9.41	44.03 ± 8.68	0.001 (1-2,3)	18.19
SBP (mm/Hg)	120.46 ± 24.66	124.26 ± 34.96	120.75 ± 15.79	0.025 (1-2)	7,41	122.2 ± 38.1	124.97 ± 16.2	128.9 ± 18.37	0.001 (1-2,3)	12.86
DBP (mm/Hg)	78.02 ± 10.69	78.74 ± 9.08	78.46 ± 8.27	0.547	1,21	78.66 ± 8.79	77.92 ± 8.64	79.34 ± 10.45	0.472	1.5
Weekday sleep (hr)	7.85 ± 1.21	8.25 ± 5.51	9.48 ± 7.96	0.078	5,10	7.92 ±4.61	8.43 ±5.53	9.95 ±8.57	0.006 (1-2,3)	10.29
Weekend sleep (hr)	8.57 ± 1.33	8.28 ± 1.89	8.23 ± 2.3	0.026 (1-2,3)	7,30	8.21 ±1.74	8.27 ±1.73	8.58 ±2.58	0.649	0.87
Framingham Risk Score	2.06 ± 7.31	0.92 ± 7.6	2.93 ± 8.14	0.019 (2-3)	8,60	MEQ* 49.68 ± 5.47	49.91 ± 6.49	47.9 ± 6.00	0.001 (3-1,2)	22.09

According to the Framingham Risk Group, the mean age of those in the low-risk group was 34.67±10.42 years, those in the moderate-risk group were 50.38±7.47 years, and those in the high-risk group were 56.92±7.79 years (p<0.05). The highest body weight and lowest height among the participants were observed in those in the high-risk group according to the Framingham Risk Score (p<0.05). The BMI value (30.61±5.71 kg/m²) of those in the low-risk group according to the Framingham Risk Score was found to be lower than the other two groups (p<0.05). FBG value was found to be 155.96±231.38 mg/dL in the low-risk group, 160.81±194.47 mg/dL in the moderate-risk group, and 174.2±229.59 mg/dL in the high-risk group (p<0.05). While the highest LDL-c level was observed in those in the high-risk group, the highest total cholesterol value was observed in those in the high-risk group (p<0.05). Sleep hours on weekdays were 8.58±2.58 hours in the high-risk group, 8.27±1.73 hours in the moderate-risk group, and 8.21±1.74 hours in the low-risk group (p<0.05). The lowest MEQ was found in individuals in the high-risk group, according to the Framingham Risk Score (p < 0.05).

Table 5 shows the relationship between age, anthropometric measurements of individuals, biochemical parameters, MEQ, and Framingham Risk Score. A positive relationship was found between the participants' ages and BMI, FBG, total cholesterol value, sleep duration, and Framingham Risk Score, and a negative relationship was found between these values ​​and MEQ. A positive relationship was found between BMI value and FBG, total cholesterol, and Framingham Risk Score. A positive relationship was found between Framingham Risk Score and age, BMI, FBG, total cholesterol, and sleep duration. A negative relationship was observed between MEQ and Framingham Risk Score (r: -0.080, p: 0.008).

**Table 5:** Relationship between anthropometric measurements of individuals, biochemical parameters, self-assessment Morningness-Eveningness Questionnaire (MEQ), and Framingham Risk Score BMI: body mass index; FBG: fasting blood glucose; *Since the data were not normally distributed, the Spearman correlation test was applied.

		Age (year)	BMI (kg/m^2^)	FBG (mg/dL)	Total cholesterol (mg/dL)	Sleep duration (sa)	Framingham Risk Score	MEQ
Age (year)	r	1						
	p	-						
BMI (kg/m^2^)	r	0.212	1					
	p	0.000	-					
FBG (mg/dL)	r	0.257	0.191	1				
	p	0.000	0.000	-				
Total cholesterol (mg/dL)	r	0.204	0.255	0.258	1			
	p	0.000	0.000	0.000	-			
Sleep duration (sa)	r	0.125	0.47	0.205	0.208	1		
	p	0.000	0.122	0.000	0.349	-		
Framingham Risk Score	r	0.798	0.267	0.340	0.388	0.130	1	
	p	0.000	0.000	0.000	0.000	0.000	-	
MEQ	r	-0.154	-0.018	-0.012	-0.053	-0.084	-0.080	1
	p	0.000	0.547	0.694	0.780	0.005	0.008	-

## Discussion

Research indicates that individuals with an evening chronotype are at a higher risk for chronic psychological and physiological diseases compared to those with a morning chronotype [20, 21, 11]. Moreover, evening chronotype individuals tend to engage more frequently in lifestyle behaviors that negatively impact health, leading to an increased prevalence of conditions such as diabetes, obesity, sleep disorders, and CVD [2, 22, 23]. In particular, the misalignment between endogenous circadian rhythms and social obligations exacerbates the risk of CVD [11]. While previous studies have examined the relationship between chronotype and various health risks, the present study offers a novel contribution by specifically evaluating CVD risk through the Framingham Risk Score in relation to morningness-eveningness status. This approach provides a more comprehensive understanding of how chronotype influences cardiovascular health and identifies evening chronotype as a potential independent risk factor for CVD.

This study evaluates the relationship between chronotype and CVD risk, demonstrating that individuals with an evening chronotype exhibit a significantly higher risk of CVD. The highest Framingham Risk Score was recorded in the eveningness group (p = 0.019). These findings are consistent with previous research, indicating that evening chronotype is associated with an increased risk of metabolic and cardiovascular disturbances [24]. The elevated Framingham Risk Scores in evening chronotype individuals can be attributed to a combination of biological and behavioral factors. Circadian misalignment, particularly prominent in evening chronotypes, disrupts metabolic homeostasis by suppressing melatonin secretion and dysregulating clock genes. This disruption triggers metabolic disturbances such as insulin resistance, dyslipidemia, and systemic inflammation, which collectively increase the risk of CVD [25]. Additionally, individuals with an evening chronotype are more likely to engage in unhealthy behaviors such as late-night eating, physical inactivity, and insufficient sleep, further exacerbating cardiovascular risk [26].

Furthermore, the social constraints imposed by work and school schedules often force evening chronotype individuals to adopt sleep-wake patterns that are misaligned with their natural circadian preferences. This misalignment, also known as "social jet lag," has been linked to increased cardiometabolic risk [27]. In particular, disruptions in the sleep-wake cycle can lead to fluctuations in blood pressure, increased sympathetic nervous system activity, and altered hormonal regulation, all of which play a critical role in the development of hypertension and atherosclerosis [28]. Given these mechanisms, the significant association between evening chronotype and higher Framingham Risk Scores observed in this study underscores the importance of considering circadian factors in CVD risk assessment and prevention. Future research should explore targeted interventions, such as chronobiology-based lifestyle modifications, to mitigate the cardiovascular risks associated with evening chronotype.

Blood pressure follows a circadian pattern, typically reaching its nadir late at night and peaking in the middle of the day [21]. Sudden fluctuations in blood pressure are recognized as significant markers of CVD risk [28]. Sleep disorders cause irregularities in blood pressure [29]. In our study, the rate of individuals with hypertension was higher in the evening group (N=13, 12.5%) than in the morning group (N=5, 9.6%). Therefore, circadian irregularity may contribute to increased risk for hypertension.

Evening chronotype individuals, who tend to have delayed sleep schedules, are at an elevated risk for obesity due to higher consumption of snacks and unhealthy foods in the late hours [30]. This study found that the proportion of individuals with a BMI over 30 kg/m² was significantly higher in the eveningness group (N=67, 64.4%) than in the morningness group (N=27, 51.9%). Furthermore, among individuals with high Framingham Risk Scores, both the prevalence of obesity and the mean BMI values were significantly elevated (p = 0.001). Additionally, evening chronotype individuals exhibited higher FBG, total cholesterol, and LDL-c levels. A systematic review evaluating six different studies found that evening chronotype individuals had a 17% increased risk of type 2 diabetes [31]. Similarly, in a study conducted among type 2 diabetes patients, FBG levels were significantly higher in individuals with an evening chronotype [32]. The current study also found significantly higher levels of FBG, total cholesterol, and systolic blood pressure in individuals with high Framingham Risk Scores (p = 0.001). Metabolic markers serve as critical indicators of CVD risk, suggesting that circadian misalignment contributes to hypertension.

Individuals with CVD often have cardiometabolic risk factors such as high blood pressure, dyslipidemia, metabolic syndrome, and high fasting plasma glucose [33]. In this study, a positive and significant relationship was found between the Framingham Risk Score and age, BMI, FBG, and total cholesterol. FBG, whether or not there is metabolic syndrome, is directly proportional to the risk of CVD according to the Framingham Risk Score [34]. A study has shown that cholesterol is independently associated with the risk of CVD [35]. HDL-c cholesterol levels also decrease with age [36]. Another study including phase angle measurements found that BMI, FBG, total cholesterol, LDL-c, age, and waist-hip ratio increase the risk of CVD [37]. Therefore, increasing age, which is an unchangeable risk factor, and the presence of obesity, high blood glucose, and total cholesterol, which are modifiable risk factors, increase the risk of CVD by increasing the Framingham risk score.

According to the recommendations of the National Sleep Foundation, seven to nine hours of sleep is considered appropriate for young adults and adults [38]. In this study, it was observed that the sleep duration of eveningness participants exceeded nine hours, especially on weekdays. In addition, it was observed that those with high sleep duration had a negative and significantly lower MEQ score and were generally evening-type individuals. In a study, it was found that individuals with an evening chronotype slept less than morningness participants and compensated for this by sleeping more on the weekends [26]. In a study conducted by Taillard et al. (1999), it was found that the sleep requirement of eveningness participants was significantly 20 minutes more than morningness participants [39]. The mechanism of why eveningness participants slept more and why they needed more sleep should be investigated in future studies.

A cohort study conducted in the UK demonstrated that a sleep duration of seven to eight hours, absence of insomnia, and a morning chronotype were associated with a lower risk of CVD [40]. A study indicated that circadian factors should be considered in the prevention of CVD and may play an important role in atherosclerosis [21]. In eveningness types, circadian disruption is considered a possible mechanism for the direct relationship between chronotype and cardiovascular health [25, 41]. In this study, there is a negative and significant relationship between MEQ and Framingham Risk Score. Thus, we have revealed the relationship between chronotype and CVD risk.

Limitations of the study

There are several potential limitations to our study. First, we did not ask for detailed information on specific environmental and social factors that may affect chronotype (e.g., social jet lag, light exposure, shift work). Second, the BMI of the study participants was generally high, which limits the generalizability of our results. Finally, due to the cross-sectional design, causal relationships cannot be definitively established. Potential selection bias due to voluntary participation. Uncontrolled confounding factors (e.g., socioeconomic status, comorbidities, medication use). Small subgroup sizes in some chronotype categories (e.g., morningness group) may reduce statistical reliability. Future longitudinal studies should aim to clarify the causative role of chronotype in CVD development.

## Conclusions

In conclusion, this study identified a significant association between evening chronotype and an increased risk of CVD. Individuals with an evening chronotype exhibited higher prevalence rates of hypertension, elevated BMI, FBG, total cholesterol, and LDL-c levels. Moreover, aging, obesity, hyperglycemia, and dyslipidemia have been found to be factors that may lead to elevated Framingham Risk Scores and further increase the risk of CVD. Given these findings, integrating chronotype considerations into preventive health strategies may enhance CVD mitigation efforts. Implementing behavioral modification interventions tailored to eveningness-type individuals, such as optimizing sleep hygiene, promoting physical activity, and regulating dietary habits, could serve as a proactive approach to reducing chronic disease burden and improving overall cardiometabolic health.

## Appendices

Appendix A 

**Table 6:** General Information

Parameters	Responses
Gender	1) Male 2) Female
Age	………………..year
Anthropometric measurements	Body weight ………… Height ………..…… Neck circumference ………… Waist circumference ………… Hip circumference ……… FBG ……… HDL …….. LDL ……….. T.Chol ……… DBP, SBP ………………
Do you smoke?	1) Yes, I still use it 2) No, I don't use 3) I quit
If you smoke;	How long have you been using it? ……………… How many per day …………………..
History of premature CVD in first-degree relatives (for men <55 years old, for women <65 years old)	1)Yes 2) No
If you are a woman and have entered menopause, your age at menopause:	………………….
How many hours do you sleep per day on average?	Weekdays ………min, Weekend………min
Do you have any chronic disease diagnosed by a doctor?	1.No 2.Hypertension 3. Hyperlipidemia 4.Psychiatric disease 5.Respiratory system diseases 6.Digestive system diseases 7.Musculoskeletal system diseases 8.Diabetes 9.Other …………………………
For which of the following conditions are you using the medication or treatment recommended by your physician?	1.None 2.Hypertension 3.Cholesterolemia 4.Hypertriglyceridemia 5.Insulin resistance 6.Diabetes 7.Obesity 8.Urea or uric acid lowering drugs
Do you take nutritional supplements?	1) Yes 2) No
Main meal consumption status and time:	( ) Morning [time: …………..] ( ) Noon time [time: …………..] ( ) Evening [time: …………..]
Snack consumption status and time:	( ) 1st snack [time: …………..] ( ) 2nd snack [time: …………..] ( ) 3rd snack [time: …………..]

Appendix B 

**Table 7:** Morningness-Eveningness Questionnaire (MEQ) Source: [12]

Questions	Responses
What time would you get up if you were entirely free to plan your day?	1. 05:00 - 06:30 AM 2. 06:30 - 07:45 AM 3. 07:45 - 09:45 AM 4. 09:45 - 11:00 AM 5. 11:00 AM - 12:00 NOON 6. 12:00 NOON - 05:00 AM
What time would you go to bed if you were entirely free to plan your evening?	1. 8:00 - 9:00 PM 2. 9:00 - 10:15 PM 3. 10:15 PM - 12:30 AM 4. 12:30 - 1:45 AM 5. 1:45 - 3:00 AM 6. 3:00 - 8:00 PM
If there is a specific time at which you have to get up in the morning, to what extent do you depend on being woken up by an alarm clock?	1. Not at all dependent 2. Slightly dependent 3. Fairly dependent 4. Very dependent
How easy do you find it to get up in the morning (when you are not woken up unexpectedly)?	1. Not at all easy 2. Not very easy 3. Fairly easy 4. Very easy
How alert do you feel during the first half hour after you wake up in the morning?	1. Not at all alert 2. Slightly alert 3. Fairly alert 4. Very alert
How hungry do you feel during the first half-hour after you wake up in the morning?	1. Not at all hungry 2. Slightly hungry 3. Fairly hungry 4. Very hungry
During the first half-hour after you wake up in the morning, how tired do you feel?	1. Very tired 2. Fairly tired 3. Fairly refreshed 4. Very refreshed
If you have no commitments the next day, what time would you go to bed compared to your usual bedtime?	1. Seldom or never later 2. Less than one hour later 3. 1-2 hours later 4. More than two hours later
You have decided to engage in some physical exercise. A friend suggests that you do this for one hour twice a week and the best time for him is between 7:00 – 8:00 am. Bearing in mind nothing but your own internal “clock”, how do you think you would perform?	1. Would be in good form 2. Would be in reasonable form 3. Would find it difficult 4. Would find it very difficult
At what time of day do you feel you become tired as a result of need for sleep?	1. 8:00 - 9:00 PM 2. 9:00 - 10:15 PM 3. 10:15 PM- 12:45 AM 4. 12:45 - 2:00 AM 5. 2:00 - 3:00 AM
You want to be at your peak performance for a test that you know is going to be mentally exhausting and will last for two hours. You are entirely free to plan your day. Considering only your own internal “clock”, which ONE of the four testing times would you choose?	1. 8:00 AM - 10:00 AM 2. 11:00 AM - 1:00 PM 3. 3:00 PM - 5:00 PM 4. 7:00 PM - 9:00 PM
If you got into bed at 11:00 PM, how tired would you be?	1. Not at all tired 2. A little tired 3. Fairly tired 4. Very tired
For some reason you have gone to bed several hours later than usual, but there is no need to get up at any particular time the next morning. Which ONE of the following are you most likely to do?	1. Will wake up at usual time, but will NOT fall back asleep 2. Will wake up at usual time and will doze thereafter 3. Will wake up at usual time but will fall asleep again 4. Will NOT wake up until later than usual
One night you have to remain awake between 4:00 – 6:00 AM in order to carry out a night watch. You have no commitments the next day. Which ONE of the alternatives will suite you best?	1. Would NOT go to bed until watch was over 2. Would take a nap before and sleep after 3. Would take a good sleep before and nap after 4. Would sleep only before watch
You have to do two hours of hard physical work. You are entirely free to plan your day and considering only your own internal “clock” which ONE of the following time would you choose?	1. 8:00 - 10:00 AM 2. 11:00 AM - 1:00 PM 3. 3:00 PM - 5:00 PM 4. 7:00 PM - 9:00 PM
You have decided to engage in hard physical exercise. A friend suggests that you do this for one hour twice a week and the best time for him is between 10:00 – 11:00 PM. Bearing in mind nothing else but your own internal “clock” how well do you think you would perform?	1. Would be in good form 2. Would be in reasonable form 3. Would find it difficult 4. Would find it very difficult
Suppose that you can choose your own work hours. Assume that you worked a FIVE hour day (including breaks) and that your job was interesting and paid by results). Which FIVE CONSECUTIVE HOURS would you select?	1. 5 hours starting between 4:00 AM and 8:00 AM 2. 5 hours starting between 8:00 AM and 9:00 AM 3. 5 hours starting between 9:00 AM and 2:00 PM 4. 5 hours starting between 2:00 PM and 5:00 PM 5. 5 hours starting between 5:00 PM and 4:00 AM
At what time of the day do you think that you reach your “feeling best” peak?	1. 5:00 - 8:00 AM 2. 8:00 - 10:00 AM 3. 10:00 AM - 5:00 PM 4. 5:00 - 10:00 PM 5. 10:00 PM - 5:00 AM
One hears about “morning” and “evening” types of people. Which ONE of these types do you consider yourself to be?	1. Definitely a “morning” type 2. Rather more a “morning” than an “evening” type 3. Rather more an “evening” than a “morning” type 4. Definitely an “evening” type

Appendix C 

**Table 8:** Framingham Risk Score Source: [18]

Risk Factor	Score		Risk Factor	Score	
	Men	Women		Men	Women
Age			HDL- Cholesterol (mg/dl)		
<35	-1	-9	≥60	-2	-3
35-39	0	-4	50-59	0	0
40-44	1	0	45-49	0	1
45-49	2	3	35-44	1	2
50-54	3	6	<35	2	5
55-59	4	7	Systolic Blood Pressure		
60-64	5	8	<120	0	-3
65-69	6	8	120-129	0	0
70-74	7	8	130-139	1	1
Total Cholesterol (mg/dl)			140-159	2	2
<160	-3	-2	≥160	3	3
169-199	0	0	Diabetes		
200-239	1	1	No	0	0
240-279	2	2	Yes	2	4
≥280	3	3	Total Points: ………………		
Smoker					
No	0	0			
Yes	2	2			

Appendix D 

**Table 9:** Physical Activity Form Source: [19]

Questions and Responses
1. Do you exercise regularly? oYes o No
2. If you exercise regularly, how many times a week do you do 20 minutes of vigorous physical activity? oMore than 3 times o1-2 times oNever
3. If you exercise regularly, how many times a week do you do 30 minutes of moderate physical activity? oMore than 5 times o3-4 times o1-2 times oNever
